# Simultaneous scattering compensation at multiple points in multi-photon microscopy

**DOI:** 10.1364/BOE.441604

**Published:** 2021-11-09

**Authors:** Molly A. May, Kai K. Kummer, Marie-Luise Edenhofer, Jeiny Luna Choconta, Michaela Kress, Monika Ritsch-Marte, Alexander Jesacher

**Affiliations:** 1 Institute of Biomedical Physics, Medical University of Innsbruck, Müllerstraße 44, 6020 Innsbruck, Austria; 2 Institute of Physiology, Medical University of Innsbruck, Schöpfstraße 41, 6020 Innsbruck, Austria

## Abstract

The two-photon fluorescence imaging depth has been significantly improved in recent years by compensating for tissue scattering with wavefront correction. However, in most approaches the wavefront corrections are valid only over a small sample region on the order of 1 to 10 µm. In samples where most scattering structures are confined to a single plane, sample conjugate correction geometries can increase the observable field to a few tens of µm. Here, we apply a recently introduced fast converging scheme for sensor-less scattering correction termed “Dynamic Adaptive Scattering compensation Holography” (DASH) in a sample conjugate configuration with a high pixel count nematic liquid crystal spatial light modulator (LC-SLM). Using a large SLM allows us to simultaneously correct for scattering at multiple field points, which can be distributed over the entire field of view provided by the objective lens. Despite the comparably slow refresh time of LC-SLMs, we achieve correction times on the order of 10 s per field point, which we show is sufficiently fast to counteract scattering at multiple sites in living mouse hippocampal tissue slices.

## 1. Introduction

Nonlinear scanning microscopy is a powerful tool for deep tissue imaging of specific structures and activities at sub-cellular resolution. Its strength relies on the use of ultrashort pulsed lasers in the near infrared range for multi-photon signal generation, which provides intrinsic optical sectioning and robustness to photon scattering. However, the high degree of heterogeneity of complex tissues such as the mouse brain still ultimately limits the imaging depth to less than 1 mm for two-photon [1] and less than 2 mm for three-photon excitation [2], thus preventing researchers from gaining valuable insights into the function of neuronal circuitries in deeper regions.

One approach to increase the penetration depth is the use of adaptive optics (AO) to correct for scattering processes. For cases with low-order turbidity, i.e. relatively mild aberrations where the laser focus is still identifiable [3], AO has become an established technique in biological microscopy.

The regime of multi-photon scattering, however, which turns a laser focus into an extended speckle pattern, poses serious challenges. A particular challenge for indirect or sensor-less approaches, which rely on iterative measurements of the fluorescent signal for phase retrieval, is the relatively long measurement time required to find a suitable correction pattern. To this end, we have witnessed notable progress in the last decade, enabling researchers to retrieve corrections within in a few seconds in two-photon fluorescence microscopy [4–7].

Another important challenge is to extend the isoplanatic patch (IP), i.e. the spatial zone of the valid wavefront correction, which can be as small as a single speckle ( ≈ 1 µm) in thick scattering tissues. In analogy to astronomical AO, the use of single or even multi-conjugate spatial light modulators (SLM) has been investigated in numerical simulations [8,9], and several groups have experimentally demonstrated single-conjugate schemes [10–14]. Here, the idea is to image the correction phase imparted by an SLM directly into the sample plane of highest turbidity. The correction pattern is then fixed to the sample structure rather than moving with the scan beam, which can significantly enlarge the IP compared to pupil-conjugate AO. Yet, few demonstrations of sample-conjugate scattering correction in living tissues have been made, and the IP in these cases was still limited to a few tens of µm [12,14].

Here we introduce a new correction scheme called *parallel multi-point correction* (PMC) in which *Dynamic Adaptive Scattering compensation Holography* (DASH) – a recently introduced fast converging scheme for phase retrieval [15] – is implemented for the first time in a sample-conjugate configuration. Similar to previous approaches, PMC is implemented by imaging the correction phase mask into the scattering tissue so that the excitation beam scans across the SLM during imaging. PMC, however, employs a high pixel count nematic LC-SLM. The large number of pixels of this SLM allows us to measure multiple scattering compensating phase patterns at distinct points of interest and to apply the corrections simultaneously. The corrected points can be distributed within a large area of about $1.6\times 1.0\;{mm}^{2}$ in the focal plane. We demonstrate PMC on an array of samples including fixed and living mouse hippocampal tissues, simultaneously correcting for up to 6 unique sample regions over distances >0.5 mm with average signal enhancement η as high as 10 times. Here, η is defined as the factor by which the maximum two-photon excitation fluorescence (TPEF) signal in an image has increased due to the correction.

A limitation of using LC-SLMs is their comparably low refresh rate of a few 100 Hz, which limits the correction speed. This is compensated, however, by the fast convergence properties of DASH, which often allows a correction to be measured in under 10 seconds. We show that this is sufficiently fast to establish significant signal enhancement at multiple points in living mouse brain tissue.

## 2. Experiment

**Fig. 1.**
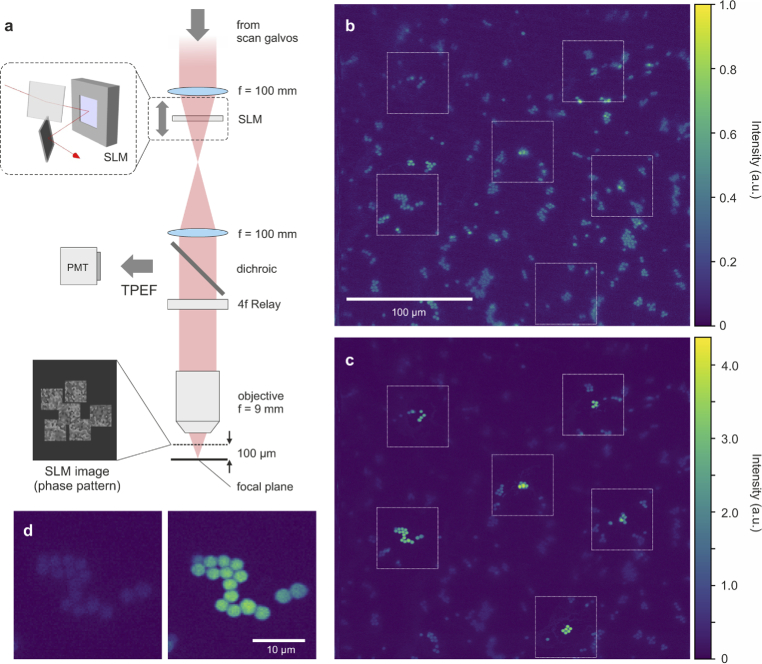
**Parallel multi-point correction experiment.** (a) In PMC, the SLM is imaged onto a user-definable layer above the focal plane and multiple corrections are simultaneously displayed. A dichroic mirror is used to separate the excitation light from the fluorescence, which is detected by a PMT. (b) Uncorrected scan image of a 4 µm bead sample through a scattering film with six regions of interest marked by white boxes. (c) Same region imaged with six simultaneously corrected points. (d) One of the six zones shown at higher magnification, before and after correction. Here, both images share the same intensity scale. Excitation wavelength = 800 nm, laser power before objective ≈50 mW, correction time ≈ 11 s per point.

Imaging was performed using a home-built scanning two-photon excitation fluorescence microscope with tunable femtosecond laser excitation (Spectra Physics Mai Tai DeepSee). A nematic liquid-crystal SLM (Meadowlark HSP1920-500-1200-HSP8, 1920 × 1152 pixels, pixel side length = 9.2 µm) was imaged onto a plane $d_{SLM}=100$ µm above the focal plane as shown in Fig. 1(a) and used to perform the scattering corrections. Next, the beam was passed through a dichroic mirror and the objective lens (Olympus XLUMPLFLN20XW, NA=1, water immersion) to the sample plane. The epi-TPEF signal was then collected and directed to a PMT (Hamamatsu H10769A-40) by the dichroic mirror. A more detailed sketch of the setup is provided in the Supplement 1.

Individual wavefront corrections were measured using DASH, which is described in detail by May et al. [15]. In summary, the incident laser beam is holographically split into a corrected field $C_{i,n}$ ( i and n representing the iteration and mode indices) representing the currently best known wavefront correction and a test wavefront $M_{n}$ , which represents the next mode to be tested, for instance (as we use in this work) a particular plane wave.

The phase pattern $\Phi_{i,n,p}$ displayed at the SLM is:

(1) $$\Phi_{i,n,p}=angle\left(\sqrt{1-f}\frac{C_{i,n}}{|C_{i,n}|}+\sqrt{f}e^{j\left(M_{n}+\varphi_{p}\right)}\right).$$

The scalar value f determines the power balance between the corrected and modulated wavefronts. It should have a value <0.5 to ensure convergence [15]. Here we use a value of f=0.2 . The TPEF signal is monitored while phase stepping the modulated beam by an offset $\varphi_{p}=p(2\pi /P)$ , where p is an integer, for a total number of phase steps P . While the minimum number of phase steps is P=3 , a value of P=5 is used here to reduce the impact of time varying noise. The phase $\phi_{i,n}$ and amplitude $a_{i,n}$ of mode $M_{n}$ are then calculated from the five TPEF signals using a phase stepping interferometry algorithm as described in [15].

Finally, the correction pattern is immediately updated by adding the measured mode with optimal amplitude and phase shift as shown in Eq. (2):

(2) $$C_{i,n+1}=C_{i,n}+a_{i,n}\;e^{j(M_{n}-\phi_{i,n})}\;.$$

This process is repeated for the desired number of modes and after each measurement the wavefront of the corrected beam improves and the signal increases. The accuracy of the phase measurements improves as the corrected wavefront is optimized, so it is often beneficial to perform multiple iterations through the test modes.

Note that while this algorithm was originally developed for a pupil-conjugate setting, its operation is functionally the same when the SLM is conjugate to any plane in the “far field” (where $d_{SLM}$ is much larger than the imaging wavelength λ ).

Because the SLM is conjugate to a plane above the focal plane, deflecting the scan mirrors displaces the beam on both the sample and the SLM. To perform PMC, a calibration must first be performed to determine the beam center position ( $x_{l},y_{l}$ ) on the SLM corresponding to field point l in the imaging plane. After calibration, PMC is implemented by positioning the beam at a point of interest in the focal plane with the scan mirrors and measuring the wavefront correction for this field point by performing DASH with a 200 × 200 pixel subset of the available SLM pixels centered on the point of interest. This process is then repeated for each desired field point and the respective correction patterns are stitched together using complex addition with Gaussian weights as shown in Eq. (3):

(3) $$\Phi_{tot}=angle\left(\sum_{l=1}^{L}e^{-({\left(x-x_{l}\right)}^{2}+{\left(y-y_{l}\right)}^{2})/(2w_{SLM}^{2})}\;e^{jangle(C_{l})}\right)\;,$$

where L denotes the total number of corrected points. The width $w_{SLM}$ of the Gaussian amplitude weighting can be freely chosen. This weighting function allows one to optimize the wavefront correction in case two phase masks overlap, because the phase of the respective stronger beam is prioritized. Here, a width $w_{SLM}=202$ µm on the SLM (corresponding to 18 µm in the sample plane) was used for all measurements.

Importantly, if the sample’s persistence time $\tau_{P}$ is short compared to the measurement time, the overall enhancement can be improved by performing a single iteration through the measurement modes at every correction point l=[1,2⋯L] before moving to the next DASH iteration. In this way, the enhancement at each field point is iteratively increased throughout the measurement, which reduces the effects of sample decorrelation.

## 3. Results

### 3.1 Parallel multi-point correction

PMC was first demonstrated on a layer of fluorescent, 4 µm polystyrene beads (Life Technologies T7284) under a scattering adhesive tape of 50 µm thickness. Further information about the tape is provided in the Supplement 1. An uncorrected image of the beads is shown in Fig. 1(b), which has a very low imaging contrast due to beam distortion by the scatterer.

DASH was then sequentially performed at six field points spread over a lateral distance of 215 µm as indicated by the white boxes in Fig. 1(b). At each point, 150 correction modes were optimized for 3 iterations. After applying the joint correction mask, a single 2-photon scan was taken. The image is shown in Fig. 1(c) and reveals improved imaging quality at all corrected field points with an average enhancement η=6 . The localized enhancement regions in this image highlight that PMC does not provide a smooth correction over the entire FOV, but rather provides enhanced “windows” through which unique sample regions can be monitored. Figure 1(d) shows one of the six zones with higher magnification, before (left) and after correction.

### 3.2 Biological Imaging

**Fig. 2.**
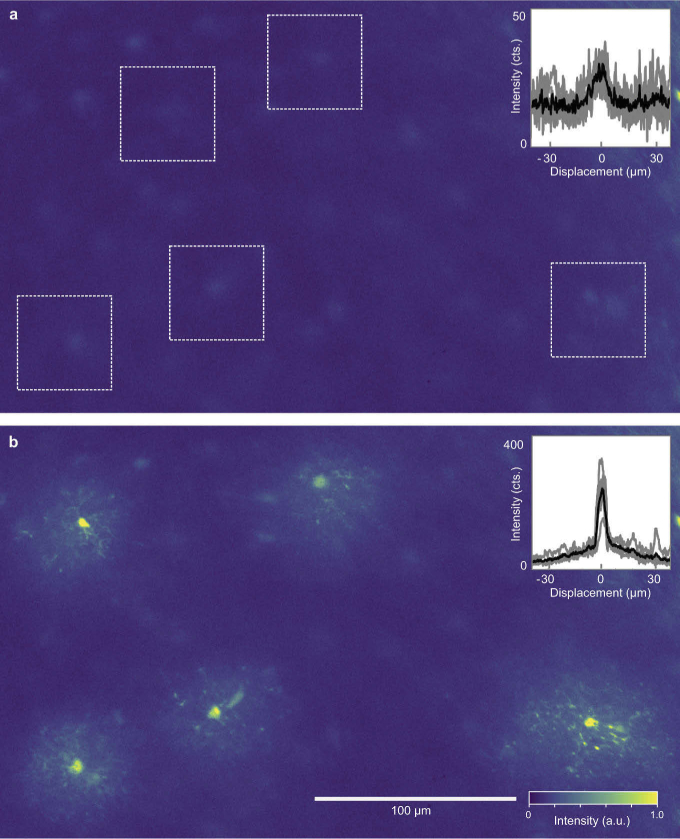
**Multi-point correction in fixed tissue at 250 µm depth.** (a) Uncorrected image of GFP expressing microglia in a fixed hippocampal tissue slice with five regions of interest marked by white boxes. (b) Corrected image where five phase masks have been applied to the regions of interest. Horizontal intensity profiles through the centers of each region before and after correction are shown as insets. Excitation wavelength = 900 nm, laser power before objective = 100 mW, correction time ≈ 7.5 s per point.

We further demonstrated the applicability of PMC by imaging GFP expressing ${\mathrm{Cx3cr1}}^{GFP}$ microglia in mouse hippocampal tissue with an excitation wavelength of 900 nm (see Supplement 1 for details of sample preparation). An uncorrected image of the hippocampal tissue at 250 µm depth is shown in Fig. 2(a) with intensity profiles from the five regions of interest marked with white boxes shown in the inset.

PMC was then performed using 100 correction modes and 3 measurement iterations at the five points. The resulting corrected image is shown in Fig. 2(b) with an average enhancement η=4.5 over a lateral range of 267 µm. Intensity profiles corresponding to those in part (a) are shown as an inset, in which bright peaks corresponding to microglia soma are surrounded by a broad corrected background region with an average IP size (FWHM) of ( 31±4 ) µm.

**Fig. 3.**
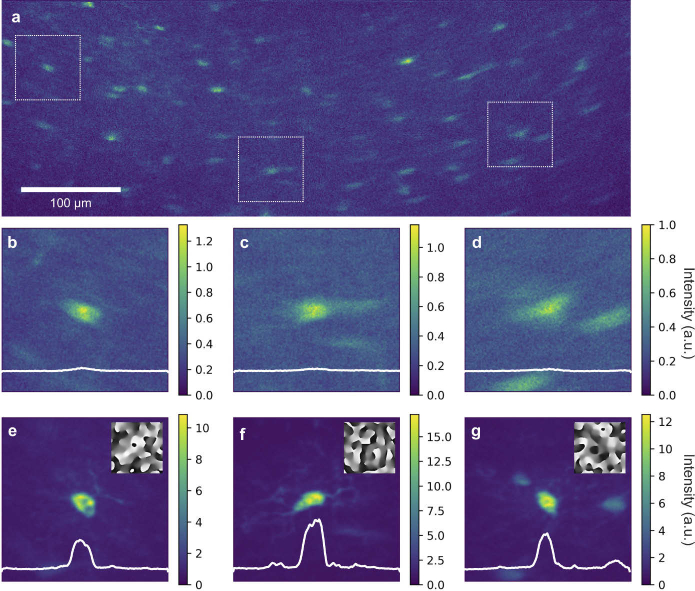
**Parallel multi-point correction at 400 µm depth.** (a) Uncorrected microglia image in fixed hippocampus with three regions of interest marked by white boxes. (b)-(d) High resolution images of the uncorrected regions of interest over 60 x 60 µm. (e)-(g) High resolution images of the region of interest with corrections applied and correction phase masks shown as insets. Horizontal intensity profiles through the centers of each region are shown by the white lines. Excitation wavelength = 900 nm, laser power before objective = 100 mW, correction time ≈ 7.5 s per point.

The imaging field was then expanded to correct three field points over ≈ 500 µm at a depth of 400 µm in the same fixed hippocampal tissue. The uncorrected image is shown in Fig. 3(a) with the regions of interest indicated by white boxes. High resolution images of the uncorrected regions of interest covering a 60 x 60 ${\mathrm{µm}}^{2}$ FOV are shown in Fig. 3(b)-(d), along with the corresponding corrected images in Fig. 3(e)-(g) with the correction masks shown as insets. Intensity profiles through the regions’ centers are shown in white, revealing an average enhancement of η=10 .

### 3.3 Live tissue imaging

Finally, we applied PMC imaging to ${\mathrm{Cx3cr1}}^{GFP}$ microglia in living mouse hippocampal tissue at a depth of 250 µm (see Supplement 1 for details of sample preparation). Interestingly, compared to fixed samples of the same type, live tissue scatters into significantly larger angles, indicating a smaller spatial scale of refractive index inhomogeneities. The number of modes was thus increased to 200 to account for the increased complexity of the scattering tissue. Further, the number of measurement iterations was decreased to 2 to minimize the measurement time.

**Fig. 4.**
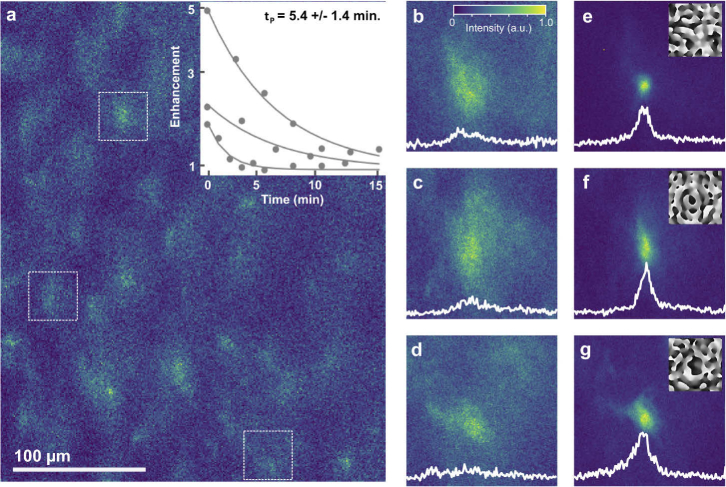
**Multi-point correction in living mouse hippocampal brain tissue.** (a) Uncorrected image of GFP stained microglia in a living hippocampus slice preparation at 250 µm depth with regions of interest marked by the white boxes. (b) - (d) High resolution images of uncorrected regions of interest over 43x43 ${\mathrm{µm}}^{2}$ . (e)-(g) High resolution images of regions of interest with three parallel corrections applied and correction phase masks shown as insets. Intensity profiles for each region are shown in white, and representative data for three persistence time measurements are shown in the inset to panel (a). Excitation wavelength = 900 nm, laser power before objective = 350 mW, correction time ≈ 25 s per point.

The full uncorrected image is shown in Fig. 4(a) and high resolution images of the three uncorrected regions of interest are shown in Fig. 4(b)-(d) extending over 43 x 43 ${\mathrm{µm}}^{2}$ . Corrected images of each region are shown in Fig. 4(e)-(g) with an average enhancement η=3.9 .

The persistence time for the corrections was also measured by fitting the time dependent enhancement to a decaying exponential $\eta (t)=\eta_{0}\;e^{-t/\tau_{P}}+1$ where $\eta_{0}$ is the enhancement at t=0 min. The persistence time was measured at nine different points on the sample with an average value of $\tau_{P}=(5.4\pm 1.4$ ) min, and several representative measurements taken at random points are shown in the inset of Fig. 4(a). This persistence time is much longer than previously measured speckle decorrelation times [16–19], in particular also compared to a similar sample [16], and might arise from the relatively slow correction measurement time of 25 s per region (this is mainly limited by the required 10 ms signal recording time per DASH measurement, which come on top of the 4 ms SLM switching time). The effects of rapidly changing sample structures were shown by Blochet et. al. [16] to average out in experiments with relatively long measurement durations, which results in lower overall enhancement but persistence times on the timescale of the measurement itself.

### 3.4 Isoplanatic patch analysis

**Fig. 5.**
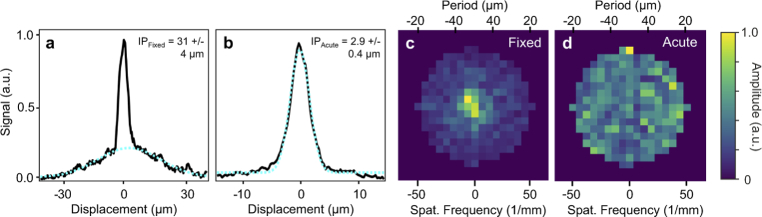
**Isoplanatic patch analysis.** Averaged intensity profiles through IPs for fixed (a) and living (b) brain imaging with representative fits (cyan, dashed). Note that the central peak in (a) arises from a cell soma and was ignored when determining the IP size. (c)-(d) Correction mode amplitudes a for fixed and living hippocampal tissue plotted as a function of spatial frequency.

The diameters (FWHM) of the IPs at 10 field points were quantified in the living hippocampal tissue with an average value of ( 2.9±0.4 ) µm. This is about an order of magnitude less than the IP sizes in fixed tissue at comparable imaging depth as highlighted in Fig. 5(a) and (b), where the average of the measured intensity profiles used to determine each IP diameter are shown in black with a representative Gaussian fit shown in cyan. Note that the narrow central peak in (a) represents the bright fluorescence contribution of a cell soma in the image and must be ignored when determining the IP size.

The mechanism behind this discrepancy was further analyzed by repeating the fixed tissue measurements with the same correction parameters and comparable imaging depth as used for living tissue (200 corrected modes, 2 DASH iterations) and plotting the modulus of the Fourier transform of the final correction mask $C_{2,200}$ to visualize the significance of each plane wave mode contribution. These distributions are shown shown in Fig. 5(c) and (d) for fixed and living tissue, respectively, revealing that the mode amplitude in the fixed tissue correction falls off sharply at period of 48 µm while in the living tissue even the highest frequency components corresponding to a period of 24 µm contributed to the correction. This indicates that the optical complexity of the fixed tissue is significantly lower than that of living tissue, which is presumably due to increased refractive index matching after transcardial perfusion of the tissue with phosphate buffered saline and paraformaldehyde.

Also, note that the size of the IP did not noticeably change over the persistence time in the living tissue (see Supplement 1), indicating that $\tau_{P}$ does not significantly depend on the structure size in this sample.

Finally, we performed numerical simulations to show the effects of various boundary conditions on the IP size. In particular, we numerically investigated the importance of an exact SLM-sample conjugation for different thicknesses of the scatterer and how the IP size is influenced by the spatial frequency distribution of its refractive index.

The scattering tissue is modelled as an absorption-free set of scattering layers with an axial interspacing of 10 µm. A scatterer of 100 µm thickness is therefore composed of 11 individual layers. The vacuum wavelength and refractive index are assumed to be 900 nm and 1.33, respectively. The axial center of the scatterer, which is also the plane of correction, is assumed to be 100 µm above the focal plane. Each scattering layer is initialized as white noise random phase scatterer and Fourier filtered using a Gaussian low-pass amplitude mask defined as $e^{-(f_{x}^{2}+f_{y}^{2})/(2\sigma_{L}^{2})}$ , where $f_{x},f_{y}$ are discrete spatial frequency coordinates and $\sigma_{L}$ defines the width of a single layer’s spatial frequency distribution. In this case, the frequency distribution of a stack of $N_{L}$ layers is given by $\sigma_{S}=\sqrt{N_{L}}\;\sigma_{L}$ .

**Fig. 6.**
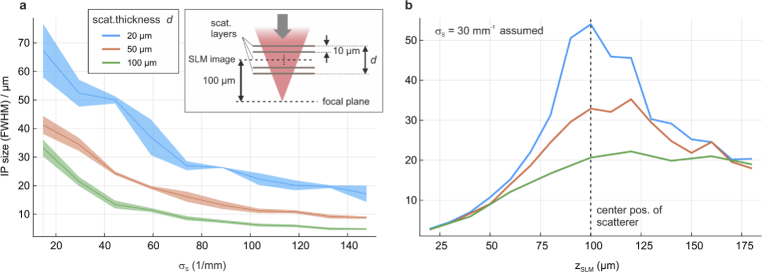
**Numerical simulation on the IP sizes in sample-conjugate DASH.** (a) The IP size shrinks with increasing spatial frequency $\sigma_{S}$ of the scatterer’s refractive index distribution. Three different scatterer thicknesses (20, 50 and 100 µm) are shown. The conceptual design of the simulation is shown in the inset. (b) Exact sample-conjugation is important for thin scattering layers, but less critical for thicker ones. Here, all scatterers are centered at the plane z = 100 µm and share the same scattering parameter $\sigma_{S}$ = 30 ${\mathrm{mm}}^{-1}$ .

The IP size is then inferred by simulating a 2D scan, assuming a 2D fluorescent layer in the focal plane, and fitting the width of the enhanced region similar to the experimental analysis. These IP sizes were then plotted for increasingly complex scatterers with higher $\sigma_{S}$ in Fig. 6(a). The IP size was additionally investigated for scatterers of 20, 50 and 100 µm thickness d , as shown in blue, red, and green, respectively. These results indicate that the IP size shrinks with both, increasing complexity and increasing thickness of the scatterer. The latter effect renders single-conjugate AO much less effective for axially extended scatterers.

When comparing these simulation results with the experimental findings of the fixed brain slice in Fig. 5(a) and (c), we find that the experimentally found values of IP size (31 µm) and scattering parameter $\sigma_{S}\approx 20\;$ ${\mathrm{mm}}^{-1}$ (found by performing a Gaussian fit to the distribution shown in Fig. 5(c)) for the fixed tissue fit well to the simulated scatterer of 100 µm thickness (green curve). Notably, the brain slice was significantly thicker with about 250 µm, but its structure may not have been as homogeneous as assumed in the model. The simulations further confirm that the IP size shrinks quickly with increasing scattering complexity, which is qualitatively also in accordance with our experimental findings.

The relevance of SLM-sample conjugation was further investigated by plotting the IP size against the SLM position for the same three scatterer thicknesses as shown in Fig. 6(b). Here, each scatterer has the same Gaussian scattering characteristics and $\sigma_{S}$ = 30 ${\mathrm{mm}}^{-1}$ . This reveals that for a 20 µm thick scatterer, an axial position mismatch of roughly 25 µm between the scatterer’s center and the SLM image (corresponding to 3 mm for the real SLM) decreases the IP size by about a factor of two. For the 50 µm scatterer, however, the IP size shrinks only slightly if the SLM image is farther from the focal plane, and for the 100 µm scatterer conjugation further from the sample has almost no effect on the IP. This circumstance allows for the implementation of an effectively conjugate scheme with an SLM in a pupil plane, as recently demonstrated for F-SHARP [14], or a plane only roughly conjugated to the scatterer as demonstrated here.

Note that for all scatterers, the IP size shrinks significantly if the SLM image moves closer to the focal plane because it is ultimately bound by the size of the displayed correction pattern, which is determined by the size of the beam cone at the SLM during the DASH measurement, which is proportional to the distance between focal plane and SLM image.

## 4. Conclusion

In summary, we have demonstrated simultaneous scattering correction at up to six field points at lateral separations of up to 0.5 mm using the previously established DASH approach in a sample-conjugate AO scheme. Applicability of the technique was demonstrated on both fixed and living microglia in mouse hippocampal tissue at depths up to 400 µm. We also established that the IPs in fixed tissue were ≈ 10 times larger than those in living tissue, which is due to the increased optical complexity of the living tissue.

The lateral separation of the corrected imaging points in PMC was limited here by the microscope FOV but it could be significantly extended for wide-field imaging experiments with a modified optical layout. Another consideration, however, is the SLM array size, which must be large enough to accommodate the desired FOV in the imaging plane. Here, our SLM extends over an area of 1.6 x 1.0 ${\mathrm{mm}}^{2}$ in the focal plane which leaves significant possibilities for increasing the correction range.

Of note, PMC is generally capable of correcting points at different depths of the specimen by adding appropriate spherical phases (“lens terms”) to the correction masks. Every correction mask could carry a different lens term. These terms will shift the laser focus to corresponding sample depths whenever the scan beam is set onto one of the corrected field points. Such an approach could be useful if individual neurons within a 3D network should be targeted by the laser.

PMC can be performed using any fluorescent molecule with excitation and emission spectra that are suitable for the microscope and also with other nonlinear processes such as second harmonic generation.

The technique has the ability to provide a good overview by increasing the accessible imaging region in turbid biological tissues by roughly an order of magnitude compared to previous conjugated scattering correction schemes. The technique is based on a sample-conjugate scheme, but not limited to correcting thin scattering layers. However, the approach provides only a set of simultaneously corrected “windows” into the tissue, not a fully corrected FOV, which makes the technique most suitable for monitoring rapid cellular activity at a set of distinct points. For example, the ability to monitor the electrical activity at several points in an extended neuronal network deep in the living brain is crucial for understanding the dynamics of neuronal circuits and how they relate to the sensations and the behavior of living organisms.

## Data Availability

Data underlying the results presented in this paper are not publicly available at this time but may be obtained from the authors upon reasonable request.

## Supplemental document

See Supplement 1 for supporting content.
