# Supplementary material for: Simultaneous scattering compensation at multiple points in multi-photon microscopy
Source: Biomed Opt Express. 2021 Nov 9;12(12):7377–87. doi: 10.1364/BOE.441604 (PMC8713664; doi:10.1364/BOE.441604)
Supplement: Supplementary file 1 [file boe-12-12-7377-s001.pdf]

Supplemental Document

Biomedical Optics EXPRESS

Simultaneous scattering compensation at multiple points in multi-photon microscopy: supplement

MOLLY A. MAY,[1] KAI K. KUMMER,[2] MARIE-LUISE EDENHOFER,[2] JEINY LUNA CHOCONTA,[2] MICHAELA KRESS,[2] MONIKA RITSCH-MARTE,[1] AND ALEXANDER JESACHER[1,*]

[1] *Institute of Biomedical Physics, Medical University of Innsbruck, Müllerstraße 44, 6020 Innsbruck, Austria*
[2] *Institute of Physiology, Medical University of Innsbruck, Schöpfstraße 41, 6020 Innsbruck, Austria*
[*] *Alexander.Jesacher@i-med.ac.at*

This supplement published with Optica Publishing Group on 9 November 2021 by The Authors under the terms of the Creative Commons Attribution 4.0 License in the format provided by the authors and unedited. Further distribution of this work must maintain attribution to the author(s) and the published article's title, journal citation, and DOI.

Supplement DOI: https://doi.org/10.6084/m9.figshare.16836685

Parent Article DOI: https://doi.org/10.1364/BOE.441604

Simultaneous scattering compensation at multiple points in multi-photon microscopy: supplemental document

This supplemental document contains additional information on the setup, the scattering tape sample, animals and tissue preparation and the temporal development of isoplanatic patch (IP) sizes in living mouse hippocampal brain slice preparations.

A. Detailed experimental setup

Figure S1 provides detailed information about our microscope setup. The SLM is mounted on a manually movable stage, together with two mirrors, which are mounted on top of each other, at different heights above the optical table. This configuration allows to vary the SLM image distance above the focal plane between 60 µm and 150 µm. The respective laser beam diameters at the SLM panel for these extremal settings are 1.1 mm and 2.8 mm. We note that the laser beam was set to underfill the objective pupil (beam diameter/pupil diameter ≈ 0.75). For a fully filled pupil the beam diameters would thus be 1.5 mm and 3.8 mm. The SLM display dimensions are 17.7×10.6 mm^2. Both galvo mirrors and the dichroic mirror are conjugate to the objective pupil.

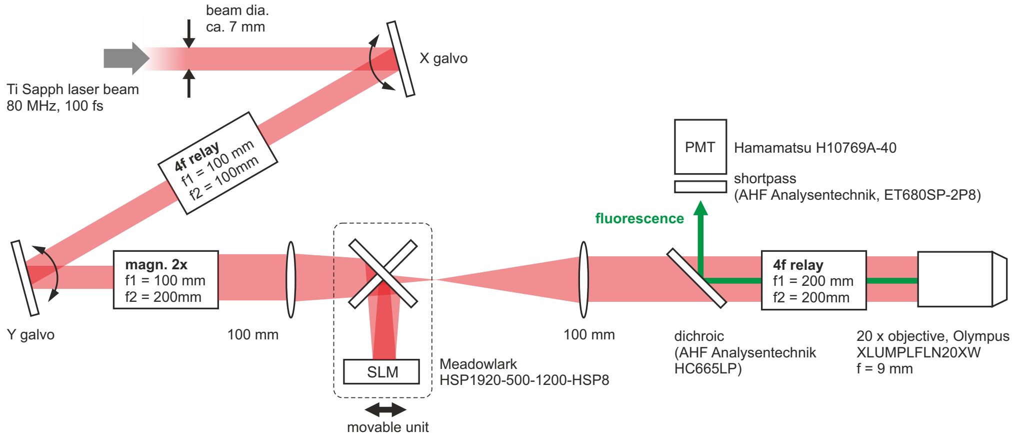

Fig. S1. Experimental setup

B. Scattering tape sample

Further information on the scattering tape sample that was used in the experiment of Fig. 1 in the main document is provided in Fig. S2. The figure shows 2-photon scan images of a thin, uniform rhodamine layer, which was covered by a 170 µm thick coverslip and a single stripe of adhesive, matte tape on top. The tape is a commercially available and has a thickness of 50 µm. Scattering is mostly occurring at the top surface of the tape. The wavefront correction comprised 400 tested plane wave modes in 3 successive iterations. The exposure time for a single measurement was 1 ms. The total correction time was about 25 seconds. We note that this correction has been performed in a pupil-AO configuration, where the SLM is imaged into the pupil of the objective lens. The size of the IP is about 10 µm.

C. Isoplanatic Patch Dynamics

The isoplanatic patch in the living tissue was monitored over the duration of the persistence time for two independent corrections as shown in Fig. S3(a)-(b). These time series show a continuous decrease in the two photon signal intensity, but no significant change in the distribution of the signal enhancement. From this, we conclude that scattering structures with spatial frequencies

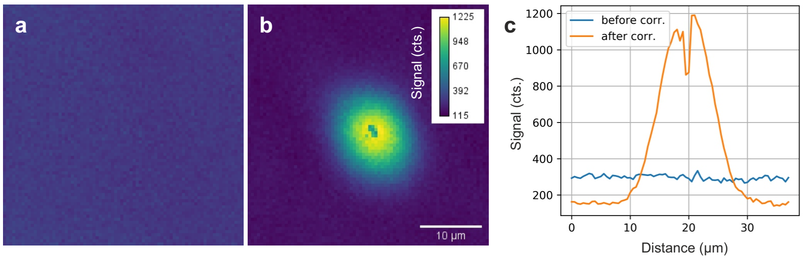

Fig. S2. **Compensating the scattering tape.** 2-photon scan images of a thin rhodamine layer underneath a coverslip plus scattering tape, before (a) and after (b) running DASH for 3 iterations including 400 plane wave modes. A 4-fold signal enhancement was achieved. (c) shows respective horizontal line profiles through the image centers. The dip in the center of the corrected patch is due to dye bleaching during the DASH measurement.

corresponding to the different corrected modes decorrelate on similar timescales. For example, if higher spatial frequency scattering structures decorrelated more quickly, the IP would grow over time even as the overall signal decreased.

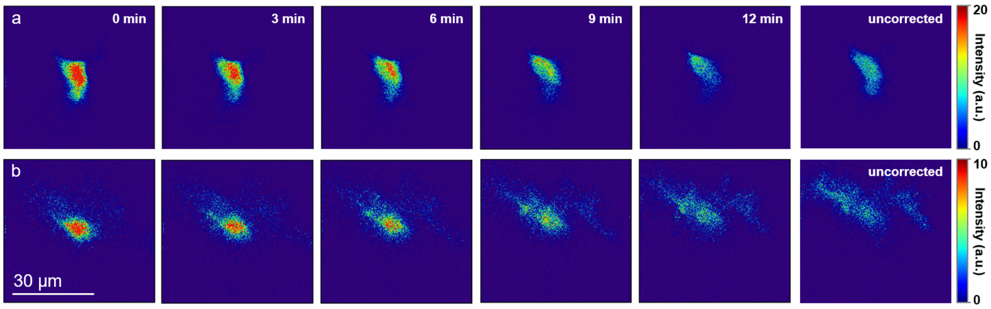

Fig. S3. **Isoplanatic patch dynamics.** (a)-(b) Time series images taken for two independent correction points over a duration of 12 min. in living microglia sample. Uncorrected images are also shown for comparison.

D. Animals and Tissue Preparation

All procedures involving animals were carried out in accordance with the Ethics Guidelines of Animal Care (Medical University of Innsbruck), as well as the European Communities Council Directive of 22 September 2010 on the protection of animals used for scientific purposes (2010/63/EU), and approved by the Austrian National Animal Experiment Ethics Committee of the Austrian Bundesministerium für Bildung, Wissenschaft und Forschung (permit number BMBWF-66.011/0148-V/3b/2019).

Mice expressing enhanced green fluorescent protein (GFP) under the promoter of Cx3cr1 (Cx3cr1^{GFP} mice, The Jackson Laboratory: 005582) were used to visualize microglial cells in fixed brain slices. Animals were housed under specific pathogen-free (SPF) conditions at constant room temperature of 24°C on a 12h light/dark cycle with lights on from 07:00 to 19:00 and had ad libitum access to autoclaved pelleted food and water.

For the fixed tissue imaging, one heterozygous female Cx3cr1$^{GFP/+}$ mouse was anesthetized with a mixture of ketamine (Ketasol®, 20 mg/ml) and xylazine (Xylasol®, 2 mg/ml) in 0.01M phosphate buffered saline (1X DPBS, 5μl/g body weight, i.p.). The mouse was transcardially perfused with 30 ml DPBS followed by 30 ml of ice-cold 4% paraformaldehyde (PFA), the brain was removed and postfixed in 4% PFA for two hours on ice. For brain slice preparation, the brain was further trimmed with a scalpel blade and glued onto the stage of a vibrating microtome (VT1000S, Leica Microsystems). Coronal slices (thickness 600 μm) containing the hippocampus were cut in PBS and subsequently stored in DPBS + 0.05% NaN_3. Slices were mounted onto microscope slides, embedded in Mowiol 4-88, coverslipped, and dried over night at room temperature.

For living tissue imaging, living coronal brain slices containing the hippocampus were prepared as previously described [1]. One heterozygous female Cx3cr1$^{GFP/+}$ mouse was anesthetized with

isoflurane (Forane®, AbbVie) and decapitated. The brain was rapidly removed and immersed in ice-cold artificial cerebrospinal fluid (aCSF) containing (in mM): NaCl 125, $NaHCO_3$ 25, D-glucose 25, KCl 2.5, NaH_2PO_4 1.25, $CaCl_2$ 2 and $MgCl_2$ 1, osmolarity: 310 mOsm/kg, pH adjusted to 7.4 with HCl [2]. The brains were trimmed with a scalpel blade and glued onto the stage of a vibrating microtome (VT1200S, Leica Microsystems). Coronal slices (thickness 600 µm) containing the hippocampus were cut in ice-cold aCSF and subsequently transferred into a slice holder for room temperature incubation in oxygenated (95% O_2, 5% CO_2) aCSF.

REFERENCES

1. K. K. Kummer, R. El Rawas, M. Kress, A. Saria, and G. Zernig, "Social interaction and cocaine conditioning in mice increase spontaneous spike frequency in the nucleus accumbens or septal nuclei as revealed by multielectrode array recordings," Pharmacology **95**, 42–49 (2015).
2. J. Bischofberger, D. Engel, L. Li, J. R. Geiger, and P. Jonas, "Patch-clamp recording from mossy fiber terminals in hippocampal slices," Nat. protocols **1**, 2075–2081 (2006).
